# The current status of gender equity in medicine in Korea: an online survey about perceived gender discrimination

**DOI:** 10.1186/s12960-020-00513-8

**Published:** 2020-10-20

**Authors:** Hyun-Young Shin, Hang Aie Lee

**Affiliations:** 1 Department of Family Medicine, Myongji Hospital, College of Medicine, Hanyang University, Seoul, Gyeonggi-do 10475 Republic of Korea; 2 Korean Medical Women’s Association, Seoul, Republic of Korea; 3 The National Assembly of The Republic of Korea, Seoul, Republic of Korea

**Keywords:** Equity, Doctor, Gender discrimination, Korea, Medical profession, Women

## Abstract

**Background:**

Although the number of women doctors has increased in South Korea, and efforts to improve gender awareness have gained importance in recent years, the issue of gender equity in the medical field has not been fully evaluated. The aim of this study was to determine the current status of gender equity in the medical profession in Korea.

**Methods:**

An online survey on perceived gender discrimination was conducted for 2 months, with both men and women doctors participating. The results were analyzed using descriptive statistics.

**Results:**

A total of 1170 doctors responded to the survey (9.2% response rate). The survey found that 47.3% of the women respondents and 18.2% of the men had experienced gender discrimination in the resident selection process (*P* < 0.05), 17.2% of the women and 8.7% of the men had experienced discrimination during the fellowship application process (*P* < 0.05), and 36.2% of the women and 8.0% of the men had experienced discrimination during the professorship application process (*P* < 0.05). Both men and women cited the issue of childbirth and parenting as the number one cause of gender discrimination against women doctors.

**Conclusions:**

This study revealed the presence of perceived gender discrimination in the Korean medical society. To address discrimination, a basic approach is necessary to change the working environment so that it is flexible for women doctors, and to change the current culture where the burden of family care, including pregnancy, childbirth, and childcare, is the primary responsibility of women.

## Background

The proportion of women doctors in Korea has increased from 12.4% in 1980 to 25.4% in 2017. It is expected to continue rising with the increase in women medical students (the proportion of women medical students was 36.0% in 2014) [1]. However, the conservative medical society proves to be a difficult environment for women doctors who face challenges when competing with their men counterparts and in being evaluated fairly. In a survey on physicians’ training and working environment conducted by the Korean Medical Association and Medical Policy Research Institute in 2017, 16.6% of women doctors and 16.0% of men doctors said there was unfairness in the selection process for the residency program [2]. The major reasons for this bias were external factors such as gender, age, and the title of the graduate school, which interfered with the resident selection process (56.8%) [2]. In a survey conducted by the Korean Women’s Medical Association in 2010, when asked whether there was gender discrimination in the resident selection process, 93.7% of women medical students answered “agree” (slightly agree, 42.8%; strongly agree, 50.9%), and 91.9% of women residents answered “agree” (slightly agree, 51.6%; strongly agree, 40.3%) [3]. In addition, in another study by the Korean Women’s Medical Association in 2011, women residents said that women doctors faced disadvantages during their employment at hospitals, with 19.6% agreeing strongly and 30.8% agreeing slightly, compared to 17.2% who disagreed slightly and 9.3% who disagreed strongly [4]. Moreover, women doctors were more likely to have the perception that they are relatively disadvantaged in the matters of professional services, senior administrative work, promotion, salary negotiations, and performance evaluation in hospitals [4].

In order for women doctors to maintain professionalism and to enhance their role in the medical field, an atmosphere of respect needs to be maintained regardless of gender, and penalties should not be imposed just because they are women. Although efforts to improve gender awareness have gained importance in recent years, the issue of gender equity in the medical field has not been fully evaluated. Therefore, the aim of this study was to identify the current status of gender equity in the medical field in Korea and to generate basic data about online survey asking perceived gender discrimination that could help establish an environment that promotes gender equity.

## Methods

### Study population

Information about Korean physicians was obtained through the databases of the Korean Medical Women’s Association (2391 persons) and the Korean Intern Resident Association (10 370 persons). The email addresses and cell phone numbers of members who agreed to receive information from their respective associations were obtained after confidentiality was assured. All participants gave their informed consent in writing. The Myongji Hospital Institutional Review Board (MJH 2018-12-003) approved this study. In order to encourage participation in the survey, the survey was uploaded on the homepage of the Korea Medical Women’s Association and the Korean Intern Resident Association websites. In addition, the survey link was sent regularly via emails and text messages to members, and relevant articles from the Korean Doctors’ Weekly were promoted. The period of the online survey was 2 months from December 2018 to January 2019.

### Variables

The questionnaire was designed to elicit the respondents’ basic information, including age and gender, their workplace (clinic, hospitals, etc.), position in the medical institution (resident, fellow, professor, paid doctor in hospital, employed in clinic, others), major department (internal medicine, surgery, others), marital status, and the number of children. The department of internal medicine included internal medicine, pediatrics, neurology, psychiatry, dermatology, rehabilitation, and family medicine. The department of surgery included urology, obstetrics/gynecology, plastic surgery, neurosurgery, ophthalmology, surgery, otorhinolaryngology, orthopedics, and thoracic surgery. The other department category included anesthesiology, radiation oncology, radiology, emergency medicine, diagnostic medicine, pathology, and nuclear medicine. Medical institution was classified into tertiary hospital, secondary hospital, hospital, clinic, and others. Marital status was classified as “agree,” “no,” and others.

### Questionnaire development and survey process

The survey questions were drafted based on consultations with advisors from the Korean Institute for Gender Equity, Promotion, and Education and the Korean Women’s Development Institute, and existing research about gender equity in the medical field. The questionnaire included questions on online survey about perceived gender discrimination encountered in the process of cultivation doctors and maintaining professionalism. The questionnaire included questions covering the whole process from entry into the resident program to appointment of fellows, and professors, employment in medical institutions, promotion, salary negotiation, and experience in the decision-making process at medical institutions (Table 1). The following is an example of the questions about gender discrimination experiences: “Did you experience gender inequity while applying for the resident program?” (the questions were designed to receive a “agree,” “disagree,” or “I don’t know” answer). Aside from the resident selection program, the questions about application for fellowship, application for professorship, employment, promotion, salary negotiations, and decision-making were asked only to specialists who have finished the resident program. A Likert rating scale (a 5-point scale from 1 to 5) was applied to the questions on “the reasons why women doctors experience gender inequity in the medical profession” (see supplementary Table 1) and “efforts to improve gender inequity in medicine.”

**Table 1:** Classification of the areas in the medical profession where gender inequity is prevalent

Classification	
Resident selection	
Fellowship application	
Professorship application	
Employment	
Salary negotiations	
Promotion	
Decision-making	
Designation of executives	
Opportunity for leadership training	

### Data analysis

Descriptive statistical analyses were performed on the results of the survey. The mean value and standard deviation of the continuous variables and the median and standard deviation of the categorical variables were calculated. A statistical analysis of the two groups (women and men) was conducted with Student’s *t* tests and chi-square tests. Statistical significance was defined as *P* < 0.05. The SAS 9.4 statistical software (SAS Institute Inc., Cary, NC) was used in this study.

## Results

Of the 12 761 people approached, 1170 responded to the survey (9.2% response rate). Among the respondents, 747 were women doctors (63.8%) and 423 were men doctors (36.2%), and by age group, 39.5% of the women respondents were aged 20–30 and 44.2% were aged 30–40, while 29.4% of the men respondents were in the 20–30 age group and 52.8% were aged 30–40. Residents were the most common category of respondents (71.5% among the women respondents and 74.0% among the men). In terms of the department, 47.5% of the women and 39.5% of the men were in internal medicine, 14.2% of the women and 23.4% of the men were in surgery, and 38.3% and 37.1% were in others. With respect to employment in medical institutions, 69.9% of the women respondents and 66.4% of the men respondents were employed in a tertiary hospital, and 20.6% and 23.4%, respectively, in a secondary hospital (Table 2).

**Table 2:** Basic characteristics of the study participants

Classification, total 1 170		Women		Men		***P*** value*
		***N***	%	***N***	%	
Gender		747	63.8	423	36.2	
Age	20–30	295	39.5	124	29.4	0.003
	30–40	330	44.2	223	52.8	
	40–50	63	8.4	46	10.9	
	50–60	43	5.8	25	5.9	
	60 ≤	16	2.1	4	1.0	
Position	Resident	534	71.5	313	74.0	0.03
	Fellow	9	1.2	11	2.6	
	Professor	119	15.9	56	13.2	
	Paid doctor in hospital	55	7.4	24	5.7	
	Employed in clinic	20	2.7	14	3.3	
	Others	10	1.3	5	1.2	
Department	Internal medicine	355	47.5	167	39.5	< 0.05
	Surgery	106	14.2	99	23.4	
	Others	286	38.3	157	37.1	
Medical institution	Tertiary hospital	522	69.9	281	66.4	0.75
	Secondary hospital	154	20.6	99	23.4	
	Hospital	23	3.1	15	3.6	
	Clinic	32	4.3	17	4.0	
	Others	16	2.1	11	2.6	
Marriage	Yes	405	54.2	190	44.9	0.01
	No	340	45.5	232	54.9	
	Others	2	0.3	1	0.2	
Number of children	0	460	67.6	233	58.3	0.02
	1	99	14.5	79	19.8	
	2	109	16.0	78	19.5	
	> 3	13	1.9	10	2.5	

*Chi-square test

**Fig. 1:**
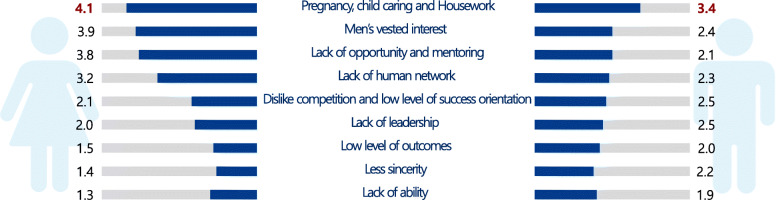
Causes of gender discrimination against women in the medical profession

In the survey, 47.3% of the women and 18.2% of the men reported having experienced gender discrimination in the resident selection process (*P* < 0.05), 17.2% of the women and 8.7% of the men said they had experienced discrimination during the fellowship application process (*P* < 0.05), and 36.2% of the women and 8.0% of the men had experienced discrimination in the professorship application process (*P* < 0.05) (Table 3). In addition, the percentage who reported gender discrimination in employment, promotion, salary negotiations, and decision-making at hospitals was 37.4%, 23.0%, 12.6%, and 21.1%, respectively, among women, and 10.0%, 5.2%, 4.1%, and 4.0%, respectively, among men (all *P* < 0.05). Both men and women cited the issue of childbirth and parenting as the number one cause of gender discrimination against women doctors (Fig. 1). Women respondents pointed out that men’s vested interests, lack of opportunity, fewer mentors, and lack of network were the other leading causes of gender discrimination against women, as these factors contributed to a men-centered environment in the medical profession. Men respondents cited women’s lack of competition, low level of success orientation, and lack of leadership as the other major causes of gender discrimination. Lack of capability, low sincerity levels, and poor performance were ranked low by both men and women (Table 4).

**Table 3:** Perceived gender discrimination in the application and promotion processes in the medical profession

Experiences in gender inequity		Women		Men		***P*** value*
		***N***	%	***N***	%	
Residency	Yes	272	47.3	62	18.2	< 0.05
	No	213	37.0	223	65.4	
	Unaware	90	15.7	56	16.4	
Fellowship	Yes	28	17.2	8	8.7	< 0.05
	No	121	74.2	79	85.9	
	Unaware	14	8.6	5	5.4	
Professorship	Yes	56	36.8	7	8.0	<0.05
	No	69	45.4	66	75.9	
	Unaware	27	17.8	14	16.1	
Employment	Yes	70	37.4	10	10.0	< 0.05
	No	88	47.1	75	75.0	
	Unaware	29	15.5	15	15.0	
Promotion	Yes	38	23.0	5	5.2	< 0.05
	No	87	52.7	75	78.1	
	Unaware	40	24.2	16	16.7	
Salary negotiations	Yes	22	12.6	4	4.1	< 0.05
	No	107	61.1	81	82.7	
	Unaware	46	26.3	13	13.3	
Decision-making	Yes	41	21.1	4	4.0	< 0.05
	No	103	53.1	84	83.2	
	Unaware	50	25.8	13	12.9	

*Student’s *t* tests

**Table 4:** Efforts to improve gender inequity in medicine

Rank	Women respondents		Men respondents	
	Reasons	Mean score (1 ~ 5)	Reasons	Mean score (1 ~ 5)
1	Setting reasonable environment for pregnancy, childbirth, and childcare	4.6 ± 0.8	Setting reasonable environment for pregnancy, childbirth, and childcare	4.0 ± 1.1
2	Correcting men-oriented medical practices pattern	4.4 ± 0.9	Improvement of the concept of gender discrimination	3.3 ± 1.3
3	Improvement of the concept of gender discrimination	4.4 ± 0.9	Strengthen women’s performance and outcomes	3.1 ± 1.3
4	Correcting gender discrimination in promotion	4.2 ± 0.9	Correcting men-oriented medical practices pattern	3.1 ± 1.3
5	Expanding women’s participation in decision-making	4.2 ± 1.0	Expanding opportunities for social network formation for women	3.0 ± 1.3
6	Expanding opportunities for social network formation for women	4.1 ± 1.0	Expanding women’s participation in decision-making	3.0 ± 1.3
7	Empowerment of management training for women	4.0 ± 1.0	Empowerment of management training for women	3.0 ± 1.3
8	Strengthen women’s performance and outcomes	3.9 ± 1.1	Correcting gender discrimination in promotion	2.9 ± 1.2

## Discussion

The study results reveal that perceived gender discrimination against women is markedly existent in the Korean medical community, with the phenomenon prevalent in all stages of employment, promotion, decision-making, and salary negotiations, as well as the resident, fellowship, and faculty recruitment process. Both women and men acknowledged that the main reason for gender discrimination is the burden of pregnancy, childbirth, and parenting on women doctors.

These results were comparable to those of previous studies such as the 2017 survey on training and work environment by the Korean Medical Association and Medical Policy Research Institute, and the Korea Women’s Medical Association studies conducted in 2010 and 2011 [2–4]. There were reports that women were relatively disadvantaged in the matters of professional duties, senior administrative work, promotion, salary negotiations, and performance evaluation in hospitals [3, 4]. In another domestic study, it was found that women doctors were struggling in a culture that was unfavorable to women, and they were in conflict with their peers, receiving negative attention from those around them during marriage and childbirth [5]. When it comes to domestic law, the “Working Standards Act” and “the Act on Supporting Gender Equity and Work-family Reconciliation” guarantee 90-day maternity leave, prohibit overtime work, offer childcare leave, allow a reduction of working hours for childcare, and provide paternity leave [6, 7]. However, in reality, there is still a gap in implementation of the abovementioned provisions in the medical field as physician resources are limited. In particular, the study stated that efforts should be made to improve the work environment for women in the medical health profession, as the law prohibits long work hours for pregnant women and bans discrimination due to childbirth [5]. Other studies have shown that women have a relatively low position in the hierarchy and accept lower authority and lower economic rewards than men. Moreover, stereotypes that men are significantly superior to women still exist [8, 9].

Many barriers to gender equity have been reported by international studies. Compensation, promotion and leadership, and academic work and recognition awards have been identified as barriers to gender equity in the medical field [10]. Above all, the most serious barrier is having children, which has been reported to be a “career stopper” in other countries. In an online survey of US physician moms, 66.3% reported having experienced gender discrimination, and 35.8% reported maternal discrimination, including at the time of pregnancy or maternity leave, and over breastfeeding [11]. Studies have reported that women doctors receive lower salaries than men, for example, women hospitalists work more but are paid less [12, 13]. Neurologists and gastroenterologists also reported that men dominate faculty positions [14, 15]. Moreover, women’s leadership role in the medical society in Japan and Europe is limited [16, 17]. To overcome these barriers, it is necessary to secure women’s leadership positions in four gatekeeper organizations—medical schools/academic medical centers, funding agencies, journals, and medical societies [18]. It has been reported that the “pipeline theory” is impossible to be realized, so a political approach is necessary, rather than a vague expectation [19]. It is also difficult for gender equity to be achieved without fundamental changes in the social role of men and women in childcare, such as offering flexible on-site childcare and part time training options [20]. Finally, regulatory measures should be applied to various approaches: cultural gender equity policies, family support policies, and active work policies at organizational, structural, and individual levels [21–23]. To implement this, a formal central registry system and a monitoring system are needed to evaluate the current state of affairs and the developments [23]. A roadmap at the regional and national level to establish policy priorities is needed to solve the problems [24, 25].

In the question related to why gender discrimination takes place in the medical field, women indicated men’s vested rights, women’s lack of leadership opportunities, and the lack of mentoring as the reasons, in that order. Female doctors in South Korea accounted for 12.4% of the total in 1980, which allowed men to obtain leadership positions more easily in the medical community [1]. In addition, in the traditional Korean consciousness that emphasizes Confucianism, there is a perception that men, with male-centered thoughts, should assume representative positions [26]. There was a practice of reserving positions for men, resulting in relatively fewer opportunities for female doctors, such as leadership roles and mentoring. This gender discrimination is similarly observed in medical institutions in other countries [27, 28]. Several programs have indeed been developed to promote women’s leadership in order to overcome this issue, with advocacy for gender equity and equality recently increasing [29, 30]. In the future, as the proportion of women in the medical field rises, it is expected that the increase in female leadership will become a method to overcome gender discrimination.

Our study has some limitations. First, the answers to the survey can be subjective, rather than objective, leading to underreporting or overreporting. Second, the response rate was low and there was a lack of information on population samples covering the whole range of participants in terms of gender, age, and regional area. Third, although the study mainly revealed recent experiences as the participants were relatively young people in their 20s, 30s, and 40s, it did not reflect the changes over time or the trends. Fourth, relatively few specialists have responded gender discrimination in the fellowship and professorship application process. Nevertheless, this study is the first survey of Korean doctors, as far as we know, that helps identify the current status of gender equity in the medical field.

## Conclusions

This study revealed the presence of gender discrimination in the Korean medical society. To address discrimination, a basic approach is necessary to monitor regularly and change the working environment so that it is flexible for women doctors, and to change the current culture where the burden of family care is the primary responsibility of women. Further large studies are needed in the future to examine gender discrimination cases across the medical community and its influencing factors.

## Supplementary information

**Additional file 1.** Supplementary Table 1.

## Data Availability

The datasets used and/or analyzed during the current study are available from the corresponding author on reasonable request.
