# Supplementary material for: The current status of gender equity in medicine in Korea: an online survey about perceived gender discrimination
Source: Hum Resour Health. 2020 Oct 20;18:78. doi: 10.1186/s12960-020-00513-8 (PMC7574171; doi:10.1186/s12960-020-00513-8)
Supplement: Supplementary file 1 — Additional file 1. Supplementary Table 1. [file 12960_2020_513_MOESM1_ESM.docx]

**Supplementary table 1.** The followings are several reasons why women doctors perceived gender discrimination in the medical profession. For each reason, please indicate the degree from 1 to 5. (score 1: very much disagree, 2: disagree, 3: neutral, 4: agree, 5: very much agree)

| **Reasons** | **Score** |
| --- | --- |
| Limited work due to women's pregnancy, childcaring, and housework |  |
| Due to the practice of the medical community, where men are holding vested interests and passing on |  |
| Women have lack of opportunity and mentoring |  |
| Women have lack social networks necessary for the medical community |  |
| Women dislike competition and have lower level of success orientation |  |
| Women have relatively lack of leadership |  |
| Women have low level of outcomes or results than men |  |
| Women have lack effort and sincerity than men |  |
| Women have lack of ability than men |  |
